# Excessive salt consumption causes systemic calcium mishandling and worsens microarchitecture and strength of long bones in rats

**DOI:** 10.1038/s41598-021-81413-2

**Published:** 2021-01-20

**Authors:** Wacharaporn Tiyasatkulkovit, Sirion Aksornthong, Punyanuch Adulyaritthikul, Pornpailin Upanan, Kannikar Wongdee, Ratchaneevan Aeimlapa, Jarinthorn Teerapornpuntakit, Catleya Rojviriya, Nattapon Panupinthu, Narattaphol Charoenphandhu

**Affiliations:** 1 Center of Calcium and Bone Research (COCAB), Faculty of Science, Mahidol University, Bangkok, 10400 Thailand; 2 Department of Biology, Faculty of Science, Chulalongkorn University, Bangkok, 10330 Thailand; 3 Department of Physiology, Faculty of Science, Mahidol University, Rama VI Road, Bangkok, 10400 Thailand; 4 Faculty of Allied Health Sciences, Burapha University, Chonburi, 20131 Thailand; 5 Department of Physiology, Faculty of Medical Science, Naresuan University, Phitsanulok, 65000 Thailand; 6 Synchrotron Light Research Institute (Public Organization), Nakhon Ratchasima, 30000 Thailand; 7 Institute of Molecular Biosciences, Mahidol University, Nakhon Pathom, 73170 Thailand; 8 The Academy of Science, The Royal Society of Thailand, Dusit, Bangkok, 10300 Thailand

**Keywords:** Bone, Bone quality and biomechanics

## Abstract

Excessive salt intake has been associated with the development of non-communicable diseases, including hypertension with several cardiovascular consequences. Although the detrimental effects of high salt on the skeleton have been reported, longitudinal assessment of calcium balance together with changes in bone microarchitecture and strength under salt loading has not been fully demonstrated. To address these unanswered issues, male Sprague–Dawley rats were fed normal salt diet (NSD; 0.8% NaCl) or high salt diet (HSD; 8% NaCl) for 5 months. Elevation of blood pressure, cardiac hypertrophy and glomerular deterioration were observed in HSD, thus validating the model. The balance studies were performed to monitor calcium input and output upon HSD challenge. The HSD-induced increase in calcium losses in urine and feces together with reduced fractional calcium absorption led to a decrease in calcium retention. With these calcium imbalances, we therefore examined microstructural changes of long bones of the hind limbs. Using the synchrotron radiation x-ray tomographic microscopy, we showed that trabecular structure of tibia and femur of HSD displayed a marked increase in porosity. Consistently, the volumetric micro-computed tomography also demonstrated a significant decrease in trabecular bone mineral density with expansion of endosteal perimeter in the tibia. Interestingly, bone histomorphometric analyses indicated that salt loading caused an increase in osteoclast number together with decreases in osteoblast number and osteoid volume. This uncoupling process of bone remodeling in HSD might underlie an accelerated bone loss and bone structural changes. In conclusion, long-term excessive salt consumption leads to impairment of skeletal mass and integrity possibly through negative calcium balance.

## Introduction

Ingestion of table salt (NaCl) in an appropriate amount is essential for the maintenance of osmolarity and volume of the extracellular fluid. However, sodium excess or deficiency can severely affect various organ functions. Current recommendation by the American Heart Association states that individuals should not consume more than 2 g of sodium per day^1^. In modern societies, however, average sodium consumption has surpassed this guideline reaching 3.4 g per day^2^. Continued salt loading gradually compromises the function of the cardiovascular and renal systems by increasing plasma osmolarity and fluid retention, thus leading to long-standing hypertension with life-threatening consequences^3^. A number of studies reported that the mechanisms underlying systemic hypertension due to high salt intake involve disarrays of neuronal and hormonal regulation. These include activation of sympathetic nervous system and renin-angiotensin system^4,5^, production of atrial natriuretic peptide^6,7^, and induction of oxidative stress^8,9^. Systemic hypertension due to high salt now emerges as a key causal factor for other metabolic diseases^10–12^.

Adverse effects of high salt diet (HSD) consumption also extend to calcium homeostasis and the regulation of bone mass at both local and systemic levels. HSD consumption significantly reduced calcium retention in adolescent girls from different ethnic backgrounds^13^. In this regard, perturbation of renal epithelial functions by excess sodium interfered with the ability to handle calcium^14^. Salt loading resulted in urinary calcium loss and increased bone turnover in postmenopausal women who already had low bone mass from estrogen deficiency^15^. Moreover, consumption of HSD upended the positive calcium balance by high calcium intake, leading to net calcium loss in postmenopausal women^16^. Concerning the gut, it has been reported that exposure to excess salt increased the number of apoptotic cells^17^, caused inflammation^18^, and leakiness^19^ of the epithelial layer. Although HSD has long been postulated to cause osteoporosis, longitudinal monitoring of the calcium balance during HSD consumption has not been fully demonstrated. Moreover, it is not known whether HSD directly affects the intestinal calcium absorption, particularly the expression of genes related to calcium transport.

Despite earlier suggestion that low bone mass was a consequence of salt-loading-induced increase in urinary calcium excretion, it remains unclear how excess salt consumption leads to aberrant bone cell functions. Addition of high concentration of NaCl directly into the culture media stimulated differentiation of osteoclasts from their precursors, i.e., mouse bone marrow macrophage and human peripheral blood mononuclear cells^20^. Interestingly, enhanced production of osteoclastogenic factors induced by HSD led to increased resorptive area and decreased bone mineral density (BMD) in long bones and vertebrae of growing male mice^21^. Mechanistically, it has been shown that an imbalance of immune interaction, i.e., T helper 17 cells and regulatory T cells, was responsible for this increase in bone resorption. However, the complete picture of bone remodeling process and microstructural changes under high salt treatment remained unexplored. In addition, data on how dietary salt affects bone strength is also lacking.

In this study, we employed a systematic approach to tackle the long-term consequence of HSD consumption on calcium handling, bone microarchitecture and strength in growing male rats. A series of calcium balance studies performed over 5 months of HSD consumption revealed mishandling of calcium in both the intestine and kidney. The present study also investigated the expression of genes related to calcium transport in the intestine and kidney. We showed that calcium loss in urine and feces compromised bone strength as determined by three-point bending tests and microarchitecture as analyzed by conventional micro-computed tomography (µCT). High-resolution synchrotron radiation x-ray tomographic microscopy (SRXTM) was also employed for better visualization of void volume and porosity in bone microstructure.

## Materials and methods

### Animals

Male Sprague–Dawley rats at 8 weeks of age were obtained from Nomura Siam International Co. Ltd. (Bangkok, Thailand). Upon arrival at the Central Animal Facility, Faculty of Science Mahidol University (MUSC-CAF, an AAALAC-accredited facility), animals were acclimatized for 7 days and maintained in stainless steel cages at 21 ± 1 °C temperature, 12/12 h light–dark cycle, and 50–60% relative humidity. The animals were fed standard chow (Perfect Companion Group Co., Ltd., Bangkok, Thailand) and reverse osmosis (RO) water ad libitum during acclimatized period. The experimental protocol was approved by the Institutional Animal Care and Use Committee (IACUC), Faculty of Science, Mahidol University. All studies related to animals were performed in accordance with relevant guidelines and regulations, including the ARRIVE guideline (http://www.ARRIVEguidelines.org).

### Experimental design

**Figure 1:**
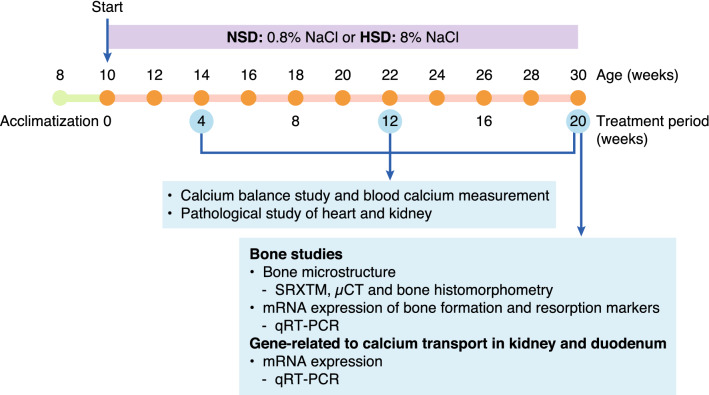
Timelines of the present experiments.

After acclimatization, body weight, systolic and diastolic blood pressure (SBP and DBP, respectively) were monitored. Non-invasive tail cuff method was used to measure SBP and DBP based on CODA tail-cuff blood pressure system (Kent Scientific Corporation, CT, USA). All rats were then randomly allocated into two groups that received normal salt diet (0.8% NaCl, NSD) or high salt diet (8% NaCl, HSD). Nutritional values of NSD (Perfect Companion Group Co., Ltd., Bangkok, Thailand) and HSD (Envigo, IN, USA) were indicated (Supplementary Table S1). There were no significant differences in the baseline values of body weight, SBP, DBP and mean arterial pressure (MAP) between two the groups (Supplementary Table S2). Normal salt and high salt containing rat chow and RO water were provided ad libitum for up to 5 months. SBP, DBP and MAP as well as body weight were recorded weekly in HSD and the corresponding age-match NSD rats throughout the experiments. Calcium balance study was performed at 1, 3 and 5 months after the random allocation. Upon euthanasia at the age of 1, 3 and 5 months in HSD and the corresponding age-match NSD rats, blood was collected by cardiac puncture to determine level of ionized calcium. Heart and kidney were collected to determine the pathological changes by histological staining. In addition, kidney and duodenum were collected to determine mRNA expression of gene related to calcium transport. Furthermore, tibiae and femurs were also collected for bone studies. The timeline of our experiments is depicted in Fig. 1.

### Histological staining and analyses

Heart and kidney were removed after euthanasia. Fresh tissue was dissected, cleaned with ice-cold saline and fixed at 4 °C with 4% paraformaldehyde for 12 h. Fixed tissues were dehydrated by graded ethanol immersion and cleared by xylene. Tissues were then embedded in paraffin and thereafter, sliced into 3-µm-thick sections. Sections from kidney and heart were stained with hematoxylin and eosin (H&E) for structural inspection. Histological analyses were examined under Olympus BX51 light microscope (Tokyo, Japan) and NIS-Elements BR Analysis 4.00 (Nikon Instruments Inc., NY, USA). Measurement of cardiac myocyte diameter was performed as previously described^22^. The diameters were obtained by measuring across the center of each cardiac myocyte at 100× magnification. The myocyte must be longitudinally cut with the presence of nuclei. For Bowman’s capsule measurement was modified from the method of Lindahl and co-workers^23^. Fifty Bowman’s capsules pictures (20× magnification) per animal were used for analysis. Total Bowman’s capsule area and glomerular tuft area were derived from tracing the outline of parietal layer and visceral epithelial layers, respectively. The difference value between total Bowman’s capsule area and glomerular tuft area was defined as Bowman’s space area.

### Calcium balance study

All animals were subjected for longitudinal assessment of calcium balance at 1, 3 and 5 months by random allocation using metabolic cages (Techniplast, Venice, Italy) to closely monitor the consumption of food and water as well as excretion of urine and feces over 3 days. Food, water, urine and feces were collected at the end of day 3, and the contents of calcium were assessed by atomic absorption spectrometer (PerkinElmer, MA, USA) as described previously^24^.

### Assessment of plasma ionized calcium

Blood samples were collected by cardiac puncture immediately after euthanasia with sterile heparinized syringe (BD Diagnostics, Plymouth, UK). Determination of ionized calcium levels was performed by using ion-selective electrodes (Nova Biomedical, Waltham, MA) under anaerobic conditions at Ramathibodi Hospital, Mahidol University, Bangkok, Thailand.

### RNA isolation

Total RNA was isolated from duodenum, kidneys and long bones of NSD and HSD rats. Rats were anesthetized and median laparotomy was performed. The duodenal segment was cut longitudinally and rinsed with cold normal saline to remove luminal contents. The mucosal cells were collected by scraping with ice-cold glass slide. For kidneys, surrounded connective tissue and renal capsule were removed and tissues were chopped into small pieces. For long bones, tissues were flushed with cold phosphate-buffered saline to remove cells in bone marrow and chopped into small pieces. Duodenal and kidney tissues were homogenized with sterile glass-Teflon Potter–Elvehjem homogenizer. Bone tissues were homogenized with Precellys Evolution Super Homogenizer using ceramic beads according to the manufacturer’s protocol (Bertin Instruments, Montigny-le-Bretonneux, France). RNA was extracted by TRIZol reagent according to the manufacturer’s protocol. The amount and purity of RNA samples were determined by NanoDrop-2000c spectrophotometer (Thermo Scientific, Waltham, MA, USA).

### Quantitative real-time RT-PCR

Synthesis of cDNA was performed by reverse transcription of one-μg total RNA with iScript cDNA synthesis kit (Bio-Rad, CA, USA). 18S rRNA (for duodenal and kidney samples) and β-actin (for long bones) were selected as housekeeping genes to evaluate consistency of the reverse transcription (i.e., variation coefficient must be less than 5%; n = 10 for each group). Quantitative real-time RT-PCR (qRT-PCR) and melting curve analyses were performed by QuantStudio 3 RT-PCR System (Applied Biosystems, CA, USA) using SsoFastEvaGreen Supermix (Bio-Rad, CA, USA). The reactions were performed for 40 cycles at 95 °C for 60 s, 51–60 °C (see annealing temperature for each primer in Supplementary Tables S3 and S4) for 30 s, and 72 °C for 30 s. All primers have been validated for specificity and efficiency by conventional RT-PCR previously^25,26^. Relative expression was calculated from the threshold cycles (C_t_) based on previously described ΔC_t_ method^27^.

### Measurement of bone microarchitecture and BMD

Bone length, three-dimensional microarchitecture and volumetric density of cortical and trabecular bone were determined by micro-computed tomography (μCT; SkyScan Model 1178, Aartselaar, Belgium) with subsequent analyses as previously described^28^. In brief, right tibia of individual rats was exposed to rotational x-ray source of 180° scanning with 0.54° angular increment using the voltage of 65 kV, the current of 615 µA and filtered by 0.5-mm aluminum. The images were reconstructed and analyzed by SkyScan CT-analyzer Version 1.11.10 (Aartselaar, Belgium).

### Bone histomorphometry

Left tibiae from NSD and HSD rats were used to determine osteoblast- and osteoclast-related parameters. After removal of adhering tissues, samples were dehydrated in serial concentrations of ethanol solutions (i.e., 70% for 3 days, 95% for 3 days and 100% for 2 days) and then embedded in methyl methacrylate resin at 42 °C for another 2 days. These resin-embedded tibiae were cut longitudinally by a tungsten carbide blade-equipped microtome (Leica, Nussloch, Germany) to obtain 7-μm-thick sections, which were stained with Goldner's trichrome method^29^ and visualized using Olympus BX51TRF light microscope (Tokyo, Japan). Analyses of tissue sections were performed using the OsteoMeasure histomorphometric system (Osteometric Inc., GA, USA). The region of interest (ROI) in this study was consistently assigned to cover the entire trabecular area of the proximal tibial metaphysis at 1–2 mm distal to the growth plate.

### Synchrotron radiation x-ray tomographic microscopy (SRXTM) of long bone

For each bone specimen, the bone was placed in a sample holder filled with cottons soaked in PBS to prevent the tissue dehydration and the displacement of the specimen during measurement. All SRXTM experiments were performed at the x-ray tomographic microscopy beamline (BL1.2W: XTM) of the Siam Photon Source (SPS), Synchrotron Light Research Institute (SLRI), Nakhon Ratchasima, Thailand. The image acquisition and parameters setup used in this experiment were based on the previous method of Tiyasatkulkovit and co-workers^30^. All tomography datasets were collected with filtered polychromatic x-ray beam at the mean energy of 10.5 keV. The x-ray projections were acquired from the detection system comprising YAG-Ce scintillator coupled microscope (OptiquePeter, France) and PCO.edge 5.5 camera (PCO Imaging, Germany). This setup yielded an effective pixel size of 3.61 × 3.61 µm^2^. The image processing and tomographic reconstruction was carried out by using Octopus Reconstruction software (Kohoutovice, Czech Republic).

As for the porosity measurement, the pore canals in the bone specimens were segmented and analyzed from the reconstructed slices by using Octopus Analysis software (Kohoutovice, Czech Republic). Herein, a fraction of the void volume over the analyzed volume (referred to as the volume of interest; VOI) were averaged from four regions in each specimen and expressed in terms of the percentage of porosity (% porosity—i.e., percent void volume). The 3D visualization and presentation of the volumetric data were rendered by using Drishti volume software (Canberra, Australia).

### Statistical analyses

The results were expressed as means ± standard error of mean (SEM). For the longitudinal study of blood pressure, which had two contributed factors (HSD and time of induction), were analyzed using two-way analysis of variance (ANOVA) with Bonferroni post-tests. For other data, the two sets of data were compared by unpaired Student’s *t*-test. The statistical significance was considered when *P* < 0.05. All data were analyzed by GraphPad Prism 7 (GraphPad, San Diego, CA, USA).

## Results

### Salt loading caused elevated blood pressure, cardiac hypertrophy and glomerular changes

**Figure 2:**
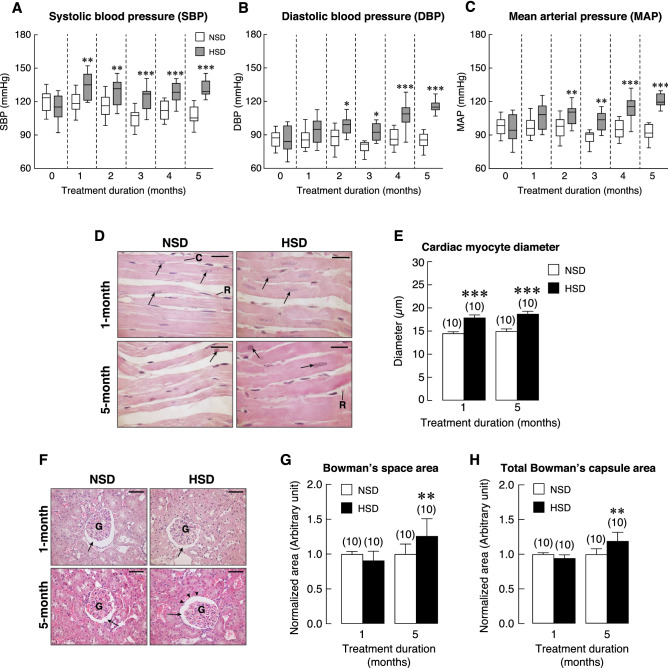
Blood pressure profile of NSD and HSD over 5-months of treatments. (**A**) Systolic blood pressure (SBP), (**B**) diastolic blood pressure (DBP) and (**C**) mean arterial pressure (MAP). (**D**) Representative H&E photomicrographs of cardiac myocytes obtained from rats fed with HSD or NSD for 1 month and 5 months. *Arrow*, nucleus of cardiac myocyte; *C*, vascular capillary; *R*, red blood cell. Scale bars, 20 μM. (**E**) Cardiac myocyte diameter of rats receiving HSD or NSD for 1 and 5 months. (**F**) Representative H&E photomicrographs of renal tissue obtained from rats fed with HSD or NSD for 1 month and 5 months. Bowman’s capsule (*arrow*) and glomerulus (*G*) were presented. Alteration of Bowman’s capsule areas was evident in HSD groups (*arrowheads*). Scale bars, 50 μm. (**G**,**H**) Quantitative analysis of the Bowman’s space and total Bowman’s capsule areas revealed an increase after the fifth month HSD consumption. Numbers of animals in each group are shown in parentheses. *P < 0.05, **P < 0.01 and ***P < 0.001 compared to period-matched NSD.

To validate our HSD model, we monitored SBP, DBP and MAP in fully conscious rats using non-invasive tail-cuff method over 5 months. At the beginning, there was no significant difference in blood pressure between the two groups. SBP in HSD group increased by the end of the first month and remained elevated throughout the experiments (Fig. 2A). Consuming HSD also increased DBP, but the effect was delayed until the end of the second month (Fig. 2B). Likewise, MAP in HSD group was gradually increased and reached statistical significance by the end of the second month (Fig. 2C). These data indicated that HSD caused elevation of SBP, DBP and MAP in rats. We next examined the consequences of high blood pressure. In general, rats received HSD remained alert and displayed normal behaviors. These rats, however, exhibited a significant decrease in body weight despite an increase in food intake (Supplementary Fig. S1A,B, respectively). Reduction in body weight rapidly developed ~ 5% (as compared to the age-matched NSD) during the first month of HSD consumption and reached ~ 10% at the fifth month. In addition, water consumption was significantly increased in HSD group (Supplementary Fig. S1C). Taken together, we found that salt loading led to hypertension with body weight loss despite increased food and water consumption.

The consequences of elevated blood pressure were examined. Since salt loading normally leads to an expansion of intravascular volume, we asked whether an increased afterload led to structural changes in the heart. It was found that HSD increased the diameters of cardiac muscle fibers within 1 month and 5 months (Fig. 2D,E). These data indicated development of cardiac hypertrophy caused by an elevated blood pressure, thus validating our model of HSD-induced hypertension. Since persistent elevation of blood pressure can damage microvascular and renal structures, we thus examined the histological changes in the kidneys after 1 month and 5 months of HSD. Here, we noticed alteration in total Bowman’s capsule area and Bowman’s space in HSD group. Quantitative analysis of the Bowman’s capsule area indeed revealed a significant increase in Bowman’s space after 5-month HSD consumption (Fig. 2F,G) consistently with a significant increase in Bowman’s capsule area (Fig. 2F,H). These data confirmed that HSD consumption led to structural changes of the renal glomeruli, which could be an early sign of renal dysfunction in hypertension.

### HSD consumption led to calcium mishandling in the kidneys and intestine

**Figure 3:**
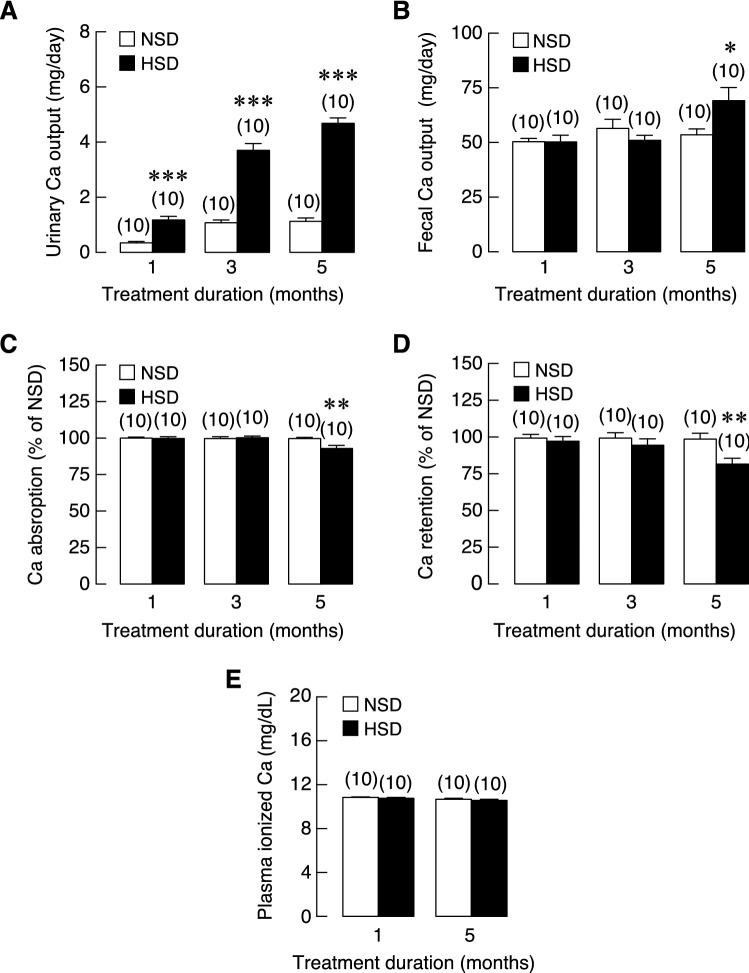
Longitudinal study of calcium balance in NSD and HSD. (**A**) Calcium output from urine, (**B**) calcium output from feces, (**C**) percentages of calcium absorption, (**D**) percentages of calcium retention, and (**E**) plasma ionized calcium. Numbers of animals in each group are shown in parentheses. *P < 0.05, **P < 0.01 and ***P < 0.001 compared to NSD.

We adopted a systematic approach to explore the effect of HSD on calcium handling. Specifically, animals were subjected to calcium balance study after 1-, 3- and 5-month of HSD consumption. Data were compared with those from rats receiving NSD at corresponding times. Focusing on the kidneys, HSD consumption led to progressive loss of calcium in the urine over 5 months (Fig. 3A). Rapid increase in calcium loss was apparent initially between time points of 1 to 3 months of HSD intake. In addition, HSD induced significant fecal calcium loss at 5 months (Fig. 3B). Next, fractional calcium absorption and calcium retention were calculated based on the results from urine and fecal outputs. We found that fractional calcium absorption was significantly decreased by 6% in HSD groups after 5 months of dietary manipulation (Fig. 3C). Reduction in calcium intake together with urinary and fecal calcium loss thus led to significant decrease in calcium retention (Fig. 3D). Nevertheless, the plasma ionized calcium remained unchanged in both groups at 1 and 5 months after dietary manipulation (Fig. 3E). In addition, it was possible that HSD-induced calcium mishandling in the kidney and intestine might have resulted from alteration at the transcriptional levels. Nevertheless, the mRNA expression levels of five candidate genes that were directly involved in the transcellular calcium transport of both kidney and intestine— i.e., TRPV5, TRPV6, S100g, PMCA_1b_ and NCX1—did not alter in HSD vs. control groups (Supplementary Figs. S2A–E for kidney and S3A–E for intestine).

### SRXTM and μCT analyses revealed microarchitectural defects in the long bones of rats receiving HSD

**Figure 4:**
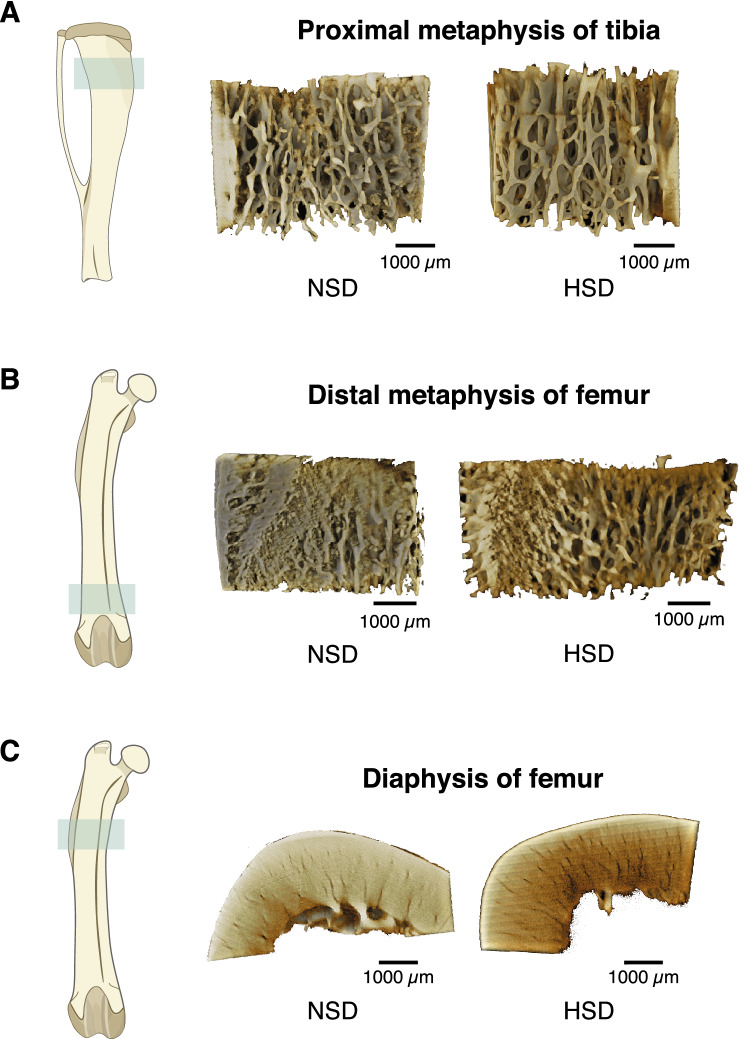
The representative images of bone specimens and their microstructures obtained from SRXTM, scale bar: 1000 µm. (**A**) Longitudinal section trabecular bone at proximal metaphysis of tibiae, (**B**) trabecular bone at distal metaphysis of femurs and (**C**) cross-sectional cortical bone at diaphysis of femurs in NSD and HSD.

**Figure 5:**
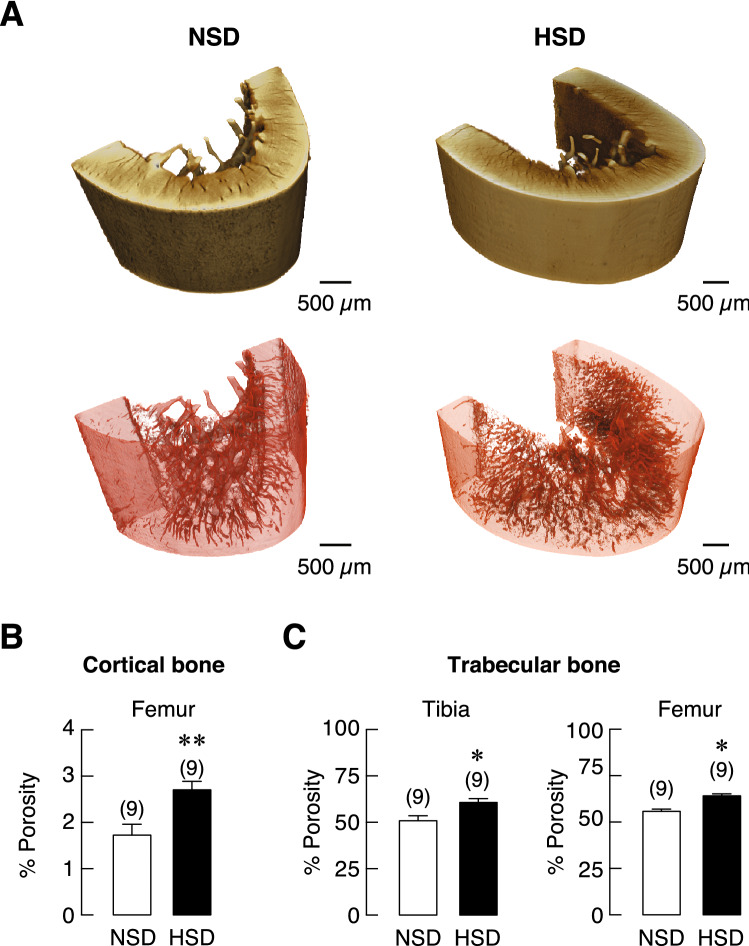
The segmentation and analysis for pore measurement in the 3D volumes of cortical bone specimens obtained from SRXTM. (**A**) 3D volume representatives of the cortical bone at diaphysis of femur from NSD and HSD group are shown in brown color gradient. The segmentation of pore canals inside the bones is rendered in red color enclosed with a transparent background for porosity measurement. All scale bars are depicted 500 µm. (**B**) quantitative analyses of the porosity of cortical bone at diaphysis of femurs and (**C**) trabecular bone at proximal metaphysis of tibiae and trabecular bone at distal metaphysis of femurs. The number of animals in each group are indicated in parentheses. *P < 0.05, **P < 0.01 compared to NSD.

Since chronic HSD consumption induced calcium loss in urine and feces, which, in turn, led to negative calcium retention, this negative impact on calcium homeostasis was expected to trigger a response by promoting calcium release from bone to maintain normal level of calcium in the blood. This highly efficient minute-to-minute regulation is possible due to the sensitive calcium-sensing receptors in the parathyroid glands. As expected, we did not detect any change in the levels of plasma ionized calcium in both groups at 1 and 5 months after dietary manipulation. We thus further hypothesized that long-term continuous release of calcium from bone could affect bone microstructure. Femurs and tibiae from rats receiving HSD or NSD for 5 months were isolated and subjected to structural and quantitative analyses using SRXTM. We examined changes in the microarchitecture of trabecular bone at metaphyses and cortical bone at midshafts. Longitudinal and cross-sectional analyses of these areas were depicted in Fig. 4A–C. Superior to those obtained from conventional μCT, the SRXTM has enable the 3D visualization of the pore networks inside the cortical bone, which contributed the porosity. As shown in Fig. 5A,B, the porosity in cortical bone, as a measurable outcome, regarding the relative volume of cortical canals was significantly increased in HSD as compared with NSD. In addition, an increase in bone porosity was also observed in the trabecular bone of tibia and femur of HSD compared with NSD (Fig. 5C).

**Figure 6:**
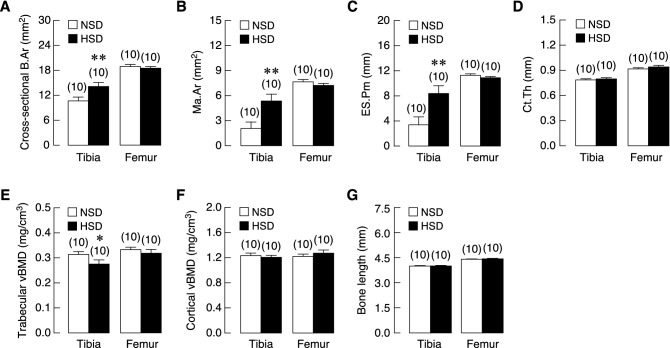
Microstructural parameters of tibiae and femurs of NSD and HSD. (**A**) Cross-sectional bone area; B.Ar, (**B**) medullary area; Ma.Ar, (**C**) endosteal perimeter; ES.Pm, (**D**) cortical thickness; Ct.Th, (**E**) trabecular volumetric BMD; vBMD, (**F**) cortical vBMD, and (**G**) bone length. Numbers of animals in each group are shown in parentheses. *P < 0.05, **P < 0.01 compared to NSD.

Next, we used conventional μCT analysis to determine geometry and BMD of long bones. HSD consumption led to a significant increase in cross-sectional area of proximal tibial metaphysis (Fig. 6A) without affecting bone length (Fig. 6G). This geometric change in HSD group prompted us to perform analyses across the surface of bone circumference of tibial metaphysis. Medullary area of tibiae metaphysis of HSD rats was markedly expanded (Fig. 6B) with a significant increase in endosteal perimeter that lined the bone marrow (Fig. 6C). We applied similar analyses to the distal metaphyses of femurs but found no significant change between HSD and NSD groups. It was also found that the circumferential areas of trabecular known as the cortical thickness was unaffected by HSD consumption (Fig. 6D). Interestingly, volumetric BMD (vBMD) of the proximal tibial metaphysis was significantly reduced in HSD groups (Fig. 6E), while there was no change in vBMD of femoral trabecular bone. Likewise, vBMD of cortical bone at both sites was unchanged (Fig. 6F). Taken together, the aforementioned data indicated that prolonged salt loading had unfavorable impacts on the long bones by increasing porosity at trabecular and cortical parts, inducing medullary expansion and decreasing trabecular BMD.

### HSD consumption disrupted the balance of bone remodeling

**Figure 7:**
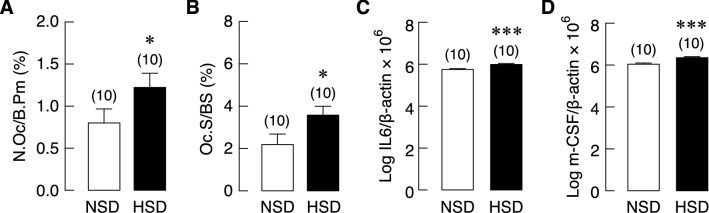
Bone histomorphometric analysis of bone resorption markers of tibiae and the mRNA expression levels of osteoblast-derived osteoclastogenic factors in long bone (humerus and radius) of NSD and HSD. (**A**) Percentages of osteoclast number per bone perimeter (N.Oc/B.Pm) and (**B**) osteoclast surface per bone surface (Oc.S/BS), and expression of (**C**) IL-6 and (**D**) m-CSF. The mRNA expression of each gene was normalized by β-actin expression. Numbers in parentheses are numbers of animals in each group. *P < 0.05, ***P < 0.001 compared to NSD.

**Figure 8:**
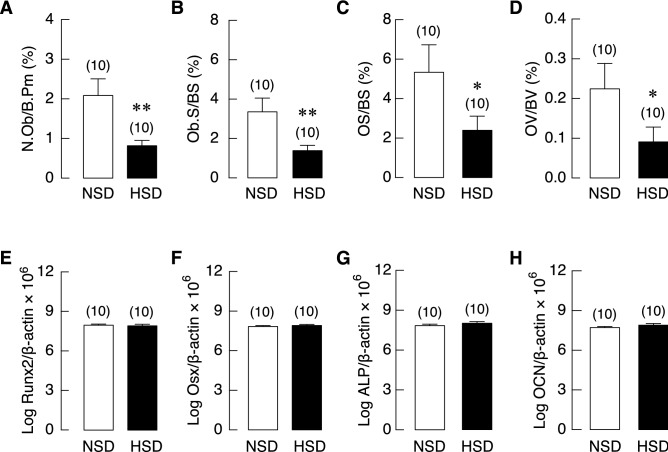
Bone histomorphometric analysis of tibiae and the mRNA expression levels of bone formation markers in long bone (humerus and radius) of NSD and HSD. (**A**) Percentages of osteoblast number per bone perimeter (N.Ob/B.Pm), (**B**) osteoblast surface per bone surface (Ob.S/BS), (**C**) osteoid surface per bone surface (OS/BS) and (**D**) osteoid volume per bone volume (OV/BV). The mRNA expression of (**E**) Runx2, (**F**) Osterix (Osx), (**G**) alkaline phosphatase (ALP) and (**H**) osteocalcin (OCN) were determined by quantitative real-time PCR and was normalized by β-actin expression. Data were analyzed by unpaired t-test. Mostly from cells of the osteoblast lineage in HSD group were similar to those in NSD controls. Numbers in parentheses are numbers of animals in each group. *P < 0.05, **P < 0.01 compared to period-matched NSD.

A decrease in trabecular vBMD had suggested that bone remodeling process was imbalance in rats with HSD treatment. We then employed bone histomorphometry to demonstrate the changes associated with bone resorption and bone formation. Regarding bone resorption, osteoclast numbers and surface were significantly increased in rats that received HSD for 5 months (Fig. 7A,B, respectively), suggesting that salt loading promoted osteoclastogenesis. Freshly isolated tibiae from HSD and NSD rats were collected for qRT-PCR to access mRNA expression of cytokine genes related to osteoclastogenesis, i.e., interleukin-6 (IL-6) and macrophage colony-stimulating factor (m-CSF). These osteoclastogenic cytokines were significantly increased in HSD group (Fig. 7C,D, respectively). Regarding bone formation, bone histomorphometry was used to visualize and quantify osteoblast number and activity from the same sets of samples. HSD consumption markedly decreased osteoblast numbers and surface (Fig. 8A,B, respectively). Moreover, we found that salt loading markedly reduced osteoid surface and volume (Fig. 8C,D, respectively). Despite the apparent changes in osteoblast number and activity, the expression levels of markers associated with osteoblast differentiation, i.e., Runx2 and osterix, as well as those associated with osteoblast activity, i.e., alkaline phosphatase and osteocalcin in HSD group were not different from NSD controls (Fig. 8E–H, respectively). These data thus indicated that HSD uncoupled bone remodeling cycle by enhancing bone resorption and suppressing bone formation.

### Salt loading caused transient mechanical defects in long bones

**Figure 9:**
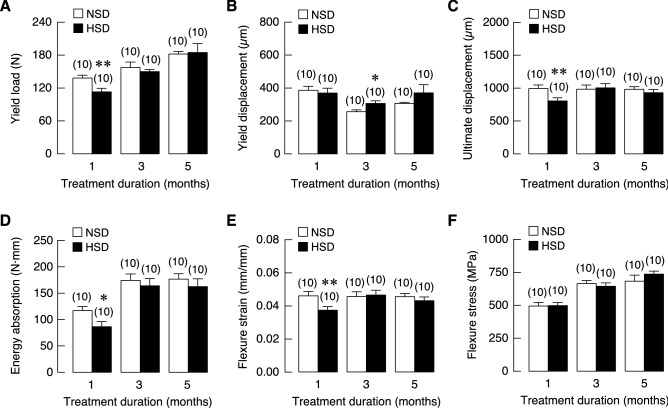
Mechanical property analysis of femurs of NSD and HSD. (**A**) Yield load, (**B**) yield displacement, (**C**) ultimate displacement, (**D**) energy absorption, (**E**) flexure strain and (**F**) flexure stress. Numbers in parentheses are numbers of animals in each group. *P < 0.05, **P < 0.01 compared to period-matched NSD.

We then turned to examine changes in the skeleton over the 5-month period. We began the process with a series of three-point bending tests to evaluate whether HSD induced cumulative mechanical defects within bone tissues. We found that HSD compromised the mechanical properties mostly after 1 month of HSD consumption. Specifically, HSD intake decreased yield load, which was the force required for the transition of elastic to plastic deformation (Fig. 9A). While there was no change in yield displacement—i.e., the bending distance prior to plastic state (Fig. 9B), HSD reduced the ultimate displacement, which was the bending distance prior to fracture (Fig. 9C), suggesting that long bones from HSD rats, once deformed, had greater tendency to break. Moreover, the capacity of energy absorption was reduced in long bones from HSD-fed rats (Fig. 9D). Consistently, flexure strain observed at the initial point of fracture was significantly reduced in HSD group (Fig. 9E). There was no change in the flexure stress observed at the initial point of fracture between two groups (Fig. 9F). Taken together, HSD led to compromised bone mechanics within the first month. However, all these mechanical changes due to salt loading disappeared in the following 3- to 5-month period (except the yield displacement at 3-month time point), suggesting that mechanical defects in bone due to salt loading were transient and correctable despite persisting calcium mishandling in chronic HSD consumption condition.

## Discussion

Herein, we demonstrated integrated findings on the effects of HSD on calcium and bone metabolism. The present dietary intervention compromised fractional calcium absorption in the gut and continuous losses of calcium in the urine, which could lead to negative consequences on the skeleton. Impaired bone strength was found after HSD consumption, whereby long bones became less resistant to external forces. Advance image analyses using SRXTM combined with conventional µCT revealed microarchitectural changes in the skeleton from HSD-fed rats. Indeed, HSD consumption further compromised bone microarchitecture and reduced vBMD. Consistently, bone histomorphometric data showed that osteoclast numbers were increased together with decreases in osteoblast numbers and osteoid volume, thus indicating an uncoupling of bone remodeling process.

Prior to studying bone microarchitectural changes, we began by validating our model of HSD-induced hypertension in rats. Early rise in salt loading-induced SBP indicated a greater sensitivity of SBP changes^31^. Complete aberration of blood pressure regulation at the fourth and the fifth month confirmed the condition of salt-induced essential hypertension. It was evident that HSD-fed rats exhibited a significant weight loss despite marked increase in food intake. Likewise, this aberrant energy metabolism was reported in another HSD-induced hypertensive model—the Dahl salt-sensitive rats^32^. It has been proposed that corticosterone-induced muscle wasting occurred in the high catabolic state due to salt loading^33^. However, in humans, excessive salt consumption was usually associated with obesity, which did not fully recapitulate this condition in HSD rat models. Indeed, salt when given with sugar mixture led to systemic hypertension and obesity in male mice compared to those that received only sugar mixture^10^.

The disturbances in cardiovascular functions then led to subsequent changes in the kidneys. Normally, an optimal ratio of glomerular and Bowman’s capsule volumes implicates effective filtration^34^. However, we found an enlargement of Bowman’s capsule area developed as a response to HSD, which was likely to be a result of a massive filtration volume, a consequence of a long-standing intake of HSD. In general, the HSD-induced hypertension often causes kidney damages in animals and men. For example, in spontaneously hypertensive rats, chronic HSD consumption led to severe damage of renal corpuscle, i.e., glomerular hypertrophy and loss of podocyte integrity^35^. Moreover, depletion of podocytes in the glomeruli was implicated in specimens from patients with hypertension^36^. Herein, we propose that Bowman’s area enlargement may be included as another critical indicator of progressive glomerular changes due to chronic HSD consumption.

Calcium concentrations in the blood and extracellular fluids are tightly controlled through the regulated translocation of calcium ions via intestinal absorption, renal reabsorption and the formation and breakdown of bone. Calcium absorption in the intestine and calcium reabsorption in the kidney involve similar processes, i.e., passive paracellular and active transcellular pathways. The transcellular pathway is a multi-carrier-mediated process, in which TRPV5 and TRPV6 allow calcium entry across the apical membrane while calbindin-D_28k_ (kidney) or calbindin-D_9k_ (intestine) translocating calcium across the cell, and finally PMCA_1b_ and NCX1 extruding calcium across the basolateral membrane. In addition, tight junction proteins, e.g., claudins, contribute to formation of ion-selective pores that mediate paracellular calcium movement^37^. The present result, however, showed no significant alteration in the mRNA levels of the calcium transport-related genes (for either paracellular passive or transcellular active transport) in the intestine and kidney. This was consistent with the previous report that showed an increased abundance of calcium transport protein calbindin-D_28k_ in HSD rats without change in the mRNA level^14^. Interestingly, HSD could alter NCX1 expression in other tissues. For example, the increased NCX1 expression on mitochondrial membrane played a pivotal role in mitochondrial calcium overload and cardiac hypertrophy induced by HSD consumption^38^. Therefore, the cellular and molecular mechanisms of how HSD consumption induced calcium mishandling in the intestine and kidney require further investigation.

Emerging evidence indicated that HSD disrupted the balance of gut microbiota that underlay the pathogenesis of hypertension^39^. Thus, it is conceivable that this HSD-induced dysbiosis compromised calcium absorption by, for example, impairing gut barrier^40^ or decreasing beneficial bacteria that normally promote calcium absorption^41,42^. Furthermore, it was proposed that disruption of gut barrier due to HSD led to early kidney injury and hypertension^19^ that could interfere with calcium reabsorption. On the other hand, consumption of the Dietary Approaches to Stop Hypertension (DASH), which contained low salt content together with high proportions of fruits, vegetables and dairy products, helped reduce the risk of urinary calcium oxalate stone formation^43^. These findings suggested that appropriate amount of salt consumption must be achieved to maintain calcium balance by intestine and kidney. Whether consumption of beneficial probiotics would counter calcium loss in HSD consumption requires more investigation. Careful examination of the host-microbe interaction in the intestine and kidneys may be helpful to uncover the mechanism of HSD-induced calcium mishandling.

Bone is a highly organized structure of calcified connective tissue and undergoes constant remodeling in a dynamic multi-step process with precise timing and coordination. Interference with any steps would likely compromise bone mass and integrity. High salt intake worsened bone microstructure along with enhancing bone loss, as seen in increased trabecular and cortical porosity and decreased trabecular vBMD in this study. Interestingly, the use of high-resolution 3D SRXTM allowed us to explore cortical bone porosity, which characterized as an important predictor of bone of fracture in human^44^. Other negative effects on bone, such as an imbalance of immune interaction (T helper 17 and regulatory T cells), have also been reported^19^. Moreover, the imbalance of calcium metabolism as well as an elevation of blood pressure might have direct influence on bone phenotype^45^. The present results that demonstrated uncoupling of bone remodeling in HSD rats, i.e., decreased formation with increased resorption, was consistent with the previous reports of HSD stimulating osteoclastogenesis and bone resorption^20,21^.

It is also possible that the accumulation of salt in bone tissue could have attracted macrophages similar to the previous findings in skin layers^46,47^. It was explained that salt deposition could alter skin microenvironment and disturb resident cells^48^. In addition, high salt concentration found in the cerebrospinal fluid led to the release of excitatory neurotransmitters, reactive oxygen species and inflammatory cytokines, which could, in turn, affect nearby cells^49^. Here, we provided evidence that HSD induced expression of osteoclastogenic factors along with increased osteoclast numbers and expansion of endosteal surface. Since deposition of sodium at the cutaneous layers was an active process that depended on the abundance of glycosaminoglycan^50^, it was plausible that excess dietary sodium could directly deposit in glycosaminoglycan-rich bone matrix and the increase in readiness of sodium in the microenvironment enhanced the migration and differentiation of osteoclast precursors. Surprisingly, although salt accumulation has been shown in bone marrow of mice fed low salt diet^51^, osteoclastogenesis was suppressed under this low salt condition. Thus, it is likely that salt-directed osteoclastogenesis and bone resorption are dependent on the readiness of sodium in bone matrix rather than the salt concentration in bone marrow.

Less is known about the effects of HSD on osteoblast functions. We demonstrated herein that rats fed HSD over 5 months had fewer osteoblasts and less matrix production. It has been shown that excess salt consumption rewired the sympathetic response causing over-activation along with an increase in blood pressure^52,53^. Since it was well established that an increase in the sympathetic activity inhibited bone formation^54^, elevation of sympathetic nerve activity after HSD could be the culprit of bone defects. On the other hand, attenuation of these signals, for example, the use of β_2_-adrenergic antagonist^55^ or endocannabinoid^56^, promoted osteoblast functions and prevented bone loss. Thus, the uses of these drugs should provide protective benefits in HSD-induced bone loss. At the cellular level, HSD did not cause any significant changes in the mRNA expression of major regulators of bone formation, i.e., Runx2 and osterix. Likewise, our previous work in spontaneous hypertensive rats also showed no change in the expression of these transcription factors^30^. Recently, salt-inducible kinase 1 (SIK1) was identified as a downstream effector for the signaling of bone morphogenetic protein (BMP)-2 in cells of the osteoblast lineage during bone formation^57^. Therefore, it would be of interest to determine the role of BMP2-SIK1 signaling axis in bone under hypertensive conditions of various etiologies.

The ultimate consequences of HSD consumption on bone fragility and the risk of fracture are not known. This study revealed for the first time that salt-induced hypertensive rats has less resistance to deformation prior to breaking in the first month of salt loading before returning to normal by the end of the fifth month. It was likely that excess salt intake had an immediate effect on bone microarchitecture and strength, which later on adapted, compensated and restored to normality. A previous study reported that bone physical property was altered in HSD-fed rats due to a decrease in heterogeneity of mineralization together with an increase in crystallinity at the cortex^21^. This uniform and highly crystalized matrix may easily permit an initiation of microcracks contributing to fracture^58,59^. Since bone calcium accretion is dependent on intestinal calcium supply in vitamin D-dependent manner^60^, the hypertension-associated dysregulation of vitamin D metabolism^61^ might aggravate bone mineralization defect and related mechanical properties (e.g., stress and yield load). Further investigations especially in human volunteers are needed to address the microarchitectural changes, deterioration of mechanical properties and their adverse consequences.

Having elaborated the negative impacts on calcium and bone metabolism caused by HSD consumption, there are some limitations needed to be addressed. The amount of dietary salt given in HSD groups was at ten times greater than those in control groups (i.e., 8% vs. 0.8% NaCl). Sodium content used in HSD group was intended to induce an obvious aberration in calcium and bone metabolism. However, such a high amount of sodium intake is unlikely to occur in human. Indeed, the sodium intake in HSD population was approximately two times greater than that in normal population^2^. Thus, it is likely that this moderate yet significant increase in salt intake is one of several factors that collectively worsens calcium metabolism and bone mass in human^15,16^.

In addition, timing and coordination of HSD-induced dysregulation of calcium and bone metabolism needed to be addressed. Here, we found that fecal calcium loss was evident only after 5 months of HSD consumption agreeing with a decrease in the intestinal calcium absorption, while urinary calcium loss and impaired bone strength was evident within the first month. It was conceivable that negative calcium balance occurred initially via urinary calcium loss. Since regulation of plasma calcium concentration was highly robust in the expense of the skeleton, this led to impaired bone resistance to the external forces by the end of the first month. Nevertheless, persistent insult of salt loading was detrimental to calcium and bone metabolism leading to increased bone porosity and decreased bone mass by the end of study. Therefore, the effects of HSD consumption on body calcium balance are highly dynamic and dependent on a number of factors. It would be interesting to incorporate dynamic tracing of calcium and sodium together with longitudinal monitoring of skeletal changes in vivo after HSD consumption.

In conclusions, our work has provided evidence on the detrimental effects of HSD on bone. Specifically, excessive salt consumption disrupts calcium balance, skeletal mass and integrity. Our approach should be beneficial in providing relevant platform and facilitating the identification of potential targets or therapeutic approach for improving bone mass and strength under prolonged high salt consumption and long-standing hypertension. Findings from our current work further establish the association of excessive salt intake, bone loss and tendency to fracture. Additional experiments in humans are required to confirm the long-lasting effects of HSD on calcium and bone metabolism.

## Supplementary Information

Supplementary Information.
