# Supplementary material for: Excessive salt consumption causes systemic calcium mishandling and worsens microarchitecture and strength of long bones in rats
Source: Sci Rep. 2021 Jan 20;11:1850. doi: 10.1038/s41598-021-81413-2 (PMC7817681; doi:10.1038/s41598-021-81413-2)
Supplement: Supplementary file 1 — Supplementary Information. [file 41598_2021_81413_MOESM1_ESM.pdf]

Supplementary Information

Excessive salt consumption causes systemic calcium mishandling and worsens microarchitecture and strength of long bones in rats

Wacharaporn Tiyasatkulkovit[1,2], Sirion Aksornthong[1,3], Punyanuch Adulyaritthikul[1,3], Pornpailin Upanan[4], Kannikar Wongdee[1,4], Ratchaneevan Aeimlapa[1,3], Jarinthorn Teerapornpuntakit[1,5], Catleya Rojviriya[6], Nattapon Panupinthu [1,3,*], Narattaphol Charoenphandhu[1,3,7,8]

[1] Center of Calcium and Bone Research (COCAB), Faculty of Science, Mahidol University, Bangkok, 10400, Thailand
[2] Department of Biology, Faculty of Science, Chulalongkorn University, Bangkok, 10330, Thailand
[3] Department of Physiology, Faculty of Science, Mahidol University, Bangkok, 10400, Thailand
[4] Faculty of Allied Health Sciences, Burapha University, Chonburi, 20131, Thailand
[5] Department of Physiology, Faculty of Medical Science, Naresuan University, Phitsanulok, 65000, Thailand
[6] Synchrotron Light Research Institute (Public Organization), Nakhon Ratchasima, 30000, Thailand
[7] Institute of Molecular Biosciences, Mahidol University, Nakhon Pathom, 73170, Thailand
[8] The Academy of Science, The Royal Society of Thailand, Dusit, Bangkok, 10300, Thailand

To whom correspondence should be addressed:

Nattapon Panupinthu, M.D., Ph.D.
Department of Physiology,
Faculty of Science, Mahidol University,
Rama VI Road, Bangkok 10400, Thailand
Tel & Fax: +66-2-201-5629
Email: nattapon.pan@mahidol.ac.th

Supplementary Table S1. Feed composition and ingredient analysis in normal-salt diet (NSD) vs. high-salt diet (HSD).

Ingredients	NSD	HSD
Calcium	1.00%	0.88%
Phosphorus	0.90%	0.64%
Magnesium	0.23%	0.21%
Manganese	171 ppm	68 ppm
Copper	22 ppm	13 ppm
Zinc	100 ppm	77 ppm
Iron	180 ppm	221 ppm
Cobalt	1.82 ppm	0.84 ppm
Selenium	0.10 ppm	0.35 ppm
Vitamin D	4,000 IU/kg	4,200 IU/kg
Sodium chloride	0.80%	8.00%
Protein	24.00%	18.50%
Fat	4.50%	5.30%
Metabolizable energy	3.04 Kcal/g	3.0 Kcal/g

Supplementary Table S2. Baseline body weight and blood pressure of 8-week-old male Sprague–Dawley rats before treated with normal-salt diet (NSD) or high-salt diet (HSD).

Group	Body weight (g)	SBP (mmHg)	DBP (mmHg)	MAP (mmHg)
NSD (n = 12)	351.9 ± 1.5	118.89 ± 2.9	85.0 ± 2.3	96.6 ± 2.4
HSD (n = 12)	353.5 ± 2.7	115.29 ± 3.1	86.5 ± 3.1	96.6 ± 3.3

Supplementary Table S3. *Rattus norvegicus* oligonucleotide sequences used in PCR experiments for studying expression of genes related to Ca^{2+} transport in the intestine and kidney.

Gene	Accession no.	Primer (forward/reverse)	Product (bp)	Annealing temperature (°C)
Genes related to Ca^{2+} transport in the intestine and kidney				
TRPV5	NM_053787	5′-CTTACGGGTTGAACACCACCA-3′<br>5′-TTGCAGAACCACAGAGCCTCTA-3′	163	60
TRPV6	NM_053686	5′-ATCCGCCGCTATGCAC-3′<br>5′-AGTTTTTCTGGTCACTGTTTTTGG-3′	80	55
S100g	X16635	5′-CCCGAAGAAATGAAGAGCATTTT-3′<br>5′-TTCTCCATCACCGTTCTTATCCA-3′	174	60
$PMCA_{1b}$	NM_053311	5′-CGCCATCTTCTGCACAATT-3′<br>5′-CAGCCATTGTTCTATTGAAAGTTC-3′	109	60
NCX1	NM_019268	5′-GTTGTGTTCGCTTGGGTTGC-3′<br>5′-CGTGGGAGTTGACTACTTTC-3′	163	55
Claudin-2	XM_236535	5′-TCTGGATGGAGTGTGCGAC-3′<br>5′-AGTGGCAAGAGGCTGGGC-3′	467	55
Housekeeping gene				
18s rRNA	DQ066896	5′-GTAACCCGTTGAACCCCATT-3′<br>5′-CCATCCAATCGGTAGTAGCG-3′	151	57

TRPV, transient receptor potential cation channel subfamily V; PMCA, plasma membrane Ca^{2+}-ATPase; NCX, sodium-calcium exchanger.

Supplementary Table S4. *Rattus norvegicus* oligonucleotide sequences used in PCR experiments for studying the expression of genes related to osteoblast differentiation and osteoblast-derived osteoclastogenic factors and housekeeping gene.

Gene	Accession no.	Primer (forward/reverse)	Product (bp)	Annealing temperature (°C)
Osteoblast differentiation markers				
Runx2	NM_053470	5′-TAACGGTCTTCACAAATCCTC-3′<br>5′-GGCGGTCAGAGAACAAACTA-3′	135	54
Osx	AY177399	5′-GCCTACTTACCCGTCTGA-3′<br>5′-CTCCAGTTGCCCACTATT-3′	139	55
ALP	NM_013059.1	5′-GCAGGATCGGAACGTCAAT-3′<br>5′-CTGGGACGGAATGGTTGAGTA-3′	144	56
OCN	X04141	5′-GAACAGACAAGTCCCACACAG-3′<br>5′-GTCTATTCACCACCTTACTGC-3′	187	60
Col1a1	NM_053304.1	5′-CAGTCGATTCACCTACAGCAC-3′<br>5′-GGGATGGAGGGAGTTTACACG-3′	194	59
Osteoblast-derived osteoclastogenic factors				
M-CSF	NM_023981	5′-ATCCAGGCAGAGACTGACAGA-3′<br>5′-CGCAGTGTAGATGAACCATCC-3′	182	55
IL-6	NM_012589	5′-GCAAGAGACTTCCAGCCAGT-3′<br>5′-AGCCTCCGACTTGTGAAGTG-3′	145	54
RANKL	NM_057149	5′-TCGCTCTGTTCCTGTACT-3′<br>5′-AGTGCTTCTGTGTCTTCG-3′	145	53
Anti-osteoclastogenic factor				
OPG	NM_012870	5′-ATTGGCTGAGTGTTCTGGT-3′<br>5′-CTGGTCTCTGTTTTGATGC-3′	140	53
Housekeeping gene				
β-actin	NM_031144	5′-CAGAGCAAGAGAGGCATCCT-3′<br>5′-GTCATCTTTTCACGGTTGGC-3′	185	54

Runx2, *runt*-related transcription factor 2; Osx, osterix; ALP, alkaline phosphatase; OCN, osteocalcin; Col1a1, collagen type 1 alpha 1; M-CSF, macrophage colony-stimulating factor; IL, interleukin; RANKL, receptor activator of nuclear factor-κB ligand; OPG, osteoprotegerin.

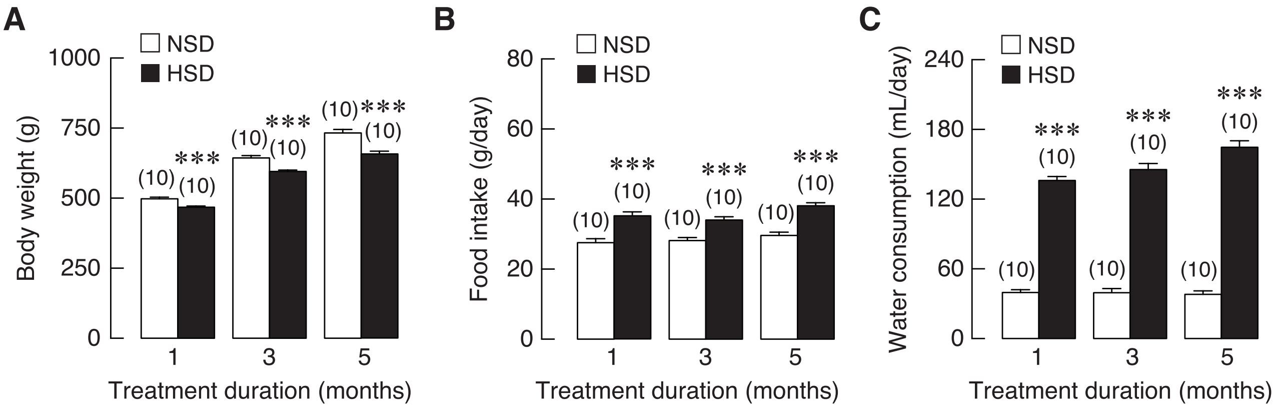

Supplementary figure S1: Tiyasatkulkovit et al.

Figure S1. (A) Body weight, (B) food intake and (C) water intake of normal salt diet (NSD)- and high salt diet (HSD)-treated groups. Data were analyzed by two-way ANOVA; ***$P < 0.001$ compared to NSD (n = 10 per group).

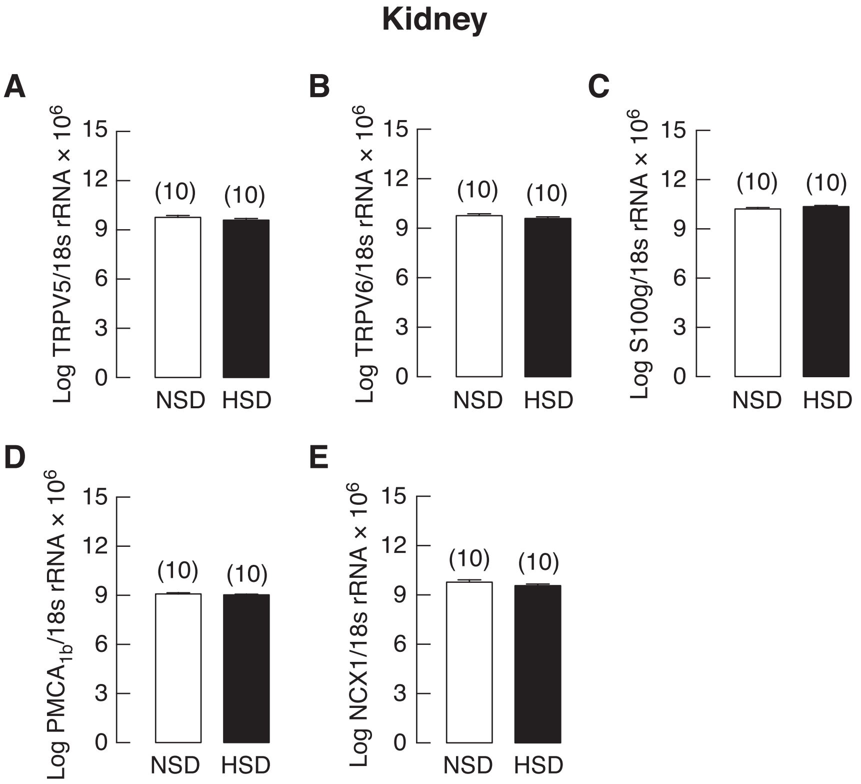

Supplementary figure S2: Tiyasatkulkovit et al.

Figure S2. The mRNA expression of gene encoding calcium transport proteins in kidney of five-month normal salt diet (NSD)- and high salt diet (HSD)-treated rats. (A) TRPV5, (B) TRPV6, (C) S100g, (D) $PMAC_{1b}$ and (E) NCX1. The mRNA expression of each gene in ten independent samples (n = 10) was determined by quantitative real-time PCR and was normalized by 18s rRNA expression. Data were analyzed by unpaired *t*-test.

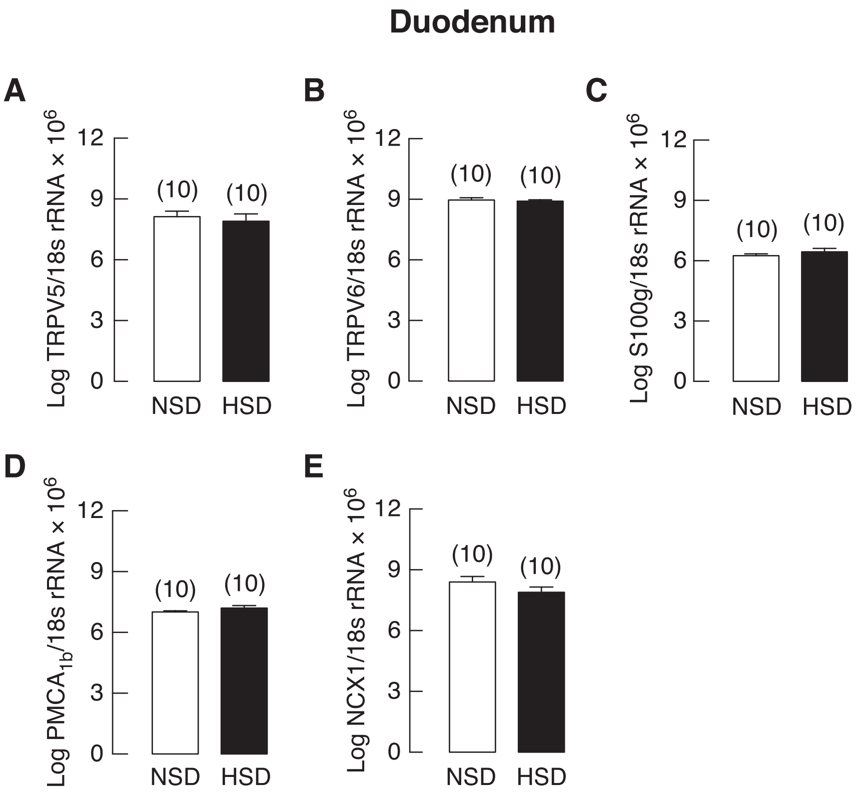

Supplementary figure S3: Tiyasatkulkovit et al.

Figure S3. The mRNA expression of gene encoding calcium transport proteins in duodenum of five-month normal salt diet (NSD)- and high salt diet (HSD)-treated rats. (A) TRPV5, (B) TRPV6, (C) S100g, (D) $PMAC_{1b}$ and (E) NCX1. The mRNA expression of each gene in ten independent samples (n = 10) was determined by quantitative real-time PCR and was normalized by 18s rRNA expression. Data were analyzed by unpaired *t*-test.
